# Effects of material properties and object orientation on precision grip kinematics

**DOI:** 10.1007/s00221-016-4631-7

**Published:** 2016-03-26

**Authors:** Vivian C. Paulun, Karl R. Gegenfurtner, Melvyn A. Goodale, Roland W. Fleming

**Affiliations:** Department of Experimental Psychology, University of Gießen, Otto-Behaghel-Str. 10F, 35394 Gießen, Germany; The Brain and Mind Institute, The University of Western Ontario, 1151 Richmond St. North, London, ON N6A 5B7 Canada

**Keywords:** Precision grip, Material, Grasping, Kinematics, Orientation

## Abstract

Successfully picking up and handling objects requires taking into account their physical properties (e.g., material) and position relative to the body. Such features are often inferred by sight, but it remains unclear to what extent observers vary their actions depending on the perceived properties. To investigate this, we asked participants to grasp, lift and carry cylinders to a goal location with a precision grip. The cylinders were made of four different materials (Styrofoam, wood, brass and an additional brass cylinder covered with Vaseline) and were presented at six different orientations with respect to the participant (0°, 30°, 60°, 90°, 120°, 150°). Analysis of their grasping kinematics revealed differences in timing and spatial modulation at all stages of the movement that depended on both material and orientation. Object orientation affected the spatial configuration of index finger and thumb during the grasp, but also the timing of handling and transport duration. Material affected the choice of local grasp points and the duration of the movement from the first visual input until release of the object. We find that conditions that make grasping more difficult (orientation with the base pointing toward the participant, high weight and low surface friction) lead to longer durations of individual movement segments and a more careful placement of the fingers on the object.

**Electronic supplementary material:**

The online version of this article (doi:10.1007/s00221-016-4631-7) contains supplementary material, which is available to authorized users.

## Introduction

Successful manual interaction with the wide range of different objects that we encounter in our environment requires highly sophisticated computations by our sensorimotor system. Even a simple two-finger pinch grip is an impressive accomplishment. Not only does our nervous system have to precisely identify the position and orientation of the object to be grasped, but also many intrinsic properties of that object must be identified before the object is lifted or even touched (Jeannerod 1981). In many cases, the only information that is available before contact is visual. Based on the visual input, the central nervous system has to prepare a motor plan that takes into account the object’s identity (e.g., a spoon serves a different purpose than a knife) and physical properties (e.g., it needs to be handled differently when made of plastic or steel). A fragile object will be lifted more delicately, while a heavy object requires a more powerful grip; rough surfaces provide higher friction and might often be grasped less carefully, while slippery surfaces challenge the system as the margin of successful grips is smaller and the position of the fingers on the object is more likely to change over time. A sponge and a wet bar of soap, for example, can be similar in terms of their shape and size, but due to their material properties they require different grips. The speed of movement and position of the effectors need to be modulated to account for differences in weight and surface properties.

The aim of this study was to systematically investigate the kinematics when subjects grasp equally sized cylinders made of different materials—thus varying in their surface properties and weight—as well as in their orientation with respect to the participant (i.e., an extrinsic property). Few studies have investigated the effects of material properties on the kinematics of grasping movements; most of them have only looked at a certain property, e.g., surface texture, in isolation or did not systematically alter extrinsic properties as well.

Theoretically, there is an almost infinite number of ways that an actor could reach for and grasp a cylinder with a precision grip in a task like ours. Indeed, the effortlessness with which we perform such actions belies the computational difficulty of selecting a single effective motor program from the manifold possibilities. Many combinations of positions of the two end effectors in space and time would lead to a successful grip. However, in practice, the way the task is accomplished is far from random, but highly stereotyped and repeatable; typical reach-to-grasp movements show a characteristic pattern. The general question for sensorimotor research on grasping is how these movements emerge: What constraints yield the specific kinematic and dynamic parameters observed in such movements.

In all likelihood, the movements are determined by several factors. First, there are certain physiological and biomechanical constraints regarding the effectors, muscles and joints participating in a certain action. In case of a precision grip this is, for example, the maximal distance between the two fingers, or the maximal force they can apply. Furthermore, there seem to be some more general principles that the central nervous system aims to accomplish in motor control, such as the smoothness of joints angle transitions (e.g., Zelik and Kuo 2012), the minimization of energy (e.g., Soechting et al. 1995) or end-state comfort (Rosenbaum et al. 1990). Other determinants are of course task-specific, i.e., here related to grasping; this also includes properties of the object to be grasped, an element that we tried to manipulate in this study. Thus, how a specific grasp is performed is influenced by many factors, each with associated costs. The sensorimotor system presumably combines these costs in some way during movement planning, and therefore in order to develop a detailed theory of motor control, all these factors must be considered. It may be possible to predict grasping kinematics using a weighted linear combination of such cost functions, similar to the prediction of grasp points on different shapes (Kleinholdermann et al. 2013). However, before such quantitative predictions can be made, parametric work is necessary to figure out which factors have an effect and how their costs combine. Here, we simultaneously varied the visually inferred material properties and orientation of objects in order to investigate how intrinsic and extrinsic object properties combine. Both factors potentially influence the speed of motion and spatial placement of the fingers at various stages during the movement. By measuring these effects simultaneously, we can constrain models of grasping and investigate how the central nervous system combines their influence.

### Related work

Weir and colleagues have investigated the effects of object weight and texture on prehension kinematics separately in two studies while keeping the other factor constant (Weir et al. 1991a, b). Weir et al. (1991b) asked participants to grasp and lift four differently weighted dowels (20, 55, 150, 410 g) with the same texture and found that the movement time was longer for larger weights. This effect seemed to be driven by larger timing differences in the post-contact phase. In a similar study, Weir et al. (1991a) asked participants to grasp three metal dowels with different textures (plain, coated with sandpaper or Vaseline) but the same weight (150 g) in a blocked fashion. Analogous to the effects of object weight, they found longer movement durations toward the slippery objects, which were mainly driven by a longer post-contact period before liftoff. However, a replication of the study with the conditions randomly interleaved, rather than blocked, showed that the visually cued texture can also have temporal consequences prior to contact (Fikes et al. 1994). Similarly, Paulun et al. (2014) found longer movement durations for a rougher (and heavier) object compared to a smoother (and lighter) object. Furthermore, they found that in the latter case, grasp points deviated more from the objects’ center and were more variable. Flatters et al. (2012) investigated qualitative movement changes for objects at different distances (10, 30, 50 cm), with different widths (50, 70, 90 mm, therefore presumably also a different weight) and surface textures (sandpaper, plastic, Vaseline). Their participants showed more ‘on-the-fly’ movements for objects with medium- or high-friction surfaces and for objects with a narrow width, i.e., movements in which the object is picked up, while the hand is in motion rather than the hand stopping to grasp and lift the object. They also found a longer movement duration for the slippery object; this effect was more pronounced in wider objects.

Fleming et al. (2002) combined weight, texture and action type in a 2 (slippery vs. non-slippery) × 2 (heavy vs. light) × 3 (grasping vs. lifting vs. posting) design, whereby trials with different weight and texture were randomly interleaved. They found that movement duration, but not reaction time (RT), was influenced by the surface texture, i.e., it was longer for the slippery object and this effect was enhanced if the object was also heavy. Texture also had an effect on RT if the task had no time constraints. More grasping errors in terms of object slips were observed for slippery or heavy objects. Differences in terms of timing of the movement could have been due to differences in planning of the movement during the approach phase or to the time needed to generate the necessary grip force once the hand had landed on the object. Because the investigators did not differentiate between pre- and post-contact phases prior to lift, they were unable to disentangle these possibilities.

Besides intrinsic factors (like weight and surface texture), grasping movements are also influenced by extrinsic object properties, such as distance, position and orientation. External factors have long been argued to only affect the transport component of the movement, whereas internal properties might only affect the grasping phase, as proposed in terms of two independent visuomotor channels (Jeannerod 1981). This view has been challenged (e.g., Smeets and Brenner 1999), and research shows that external factors can influence both the reach and grasp component. In different setups and tasks, object orientation has been shown to influence movement trajectories (Desmurget and Prablanc 1997; Fan et al. 2006; Gentilucci et al. 1996; Mamassian 1997), wrist rotation (Cuijpers et al. 2004; Desmurget and Prablanc 1997; Fan et al. 2006; Gentilucci et al. 1996; Glover and Dixon 2001; Mamassian 1997) and the orientation of the grasp axis (Chen and Saunders 2015; Kleinholdermann et al. 2013). It has also been shown that perturbations of the object’s orientation can be adjusted online (Chen and Saunders 2015; Desmurget and Prablanc 1997; Voudouris et al. 2013).

In the present study, we systematically investigated the influence of the object’s orientation in the horizontal plane on the kinematics of a two-finger precision grip. Besides orientation, we also varied the material of the objects to be grasped, i.e., weight and surface properties were altered simultaneously as is mostly the case in our natural environment. We were interested in the individual effects of each factor, material and orientation, as well as potential interactions between these intrinsic and extrinsic object properties on prehension. For a better understanding of the underlying mechanisms, we measured the placement of the fingers on the object and divided the executed movements into distinct segments in order to analyze the timing of the movement precisely. We thus aimed to add to existing literature by (1) manipulating material properties in a more natural manner (e.g., by varying surface properties and weight simultaneously) and (2) combining this manipulation of intrinsic properties with variations in an extrinsic property (orientation) and (3) measuring the effects in both the spatial as well as the temporal domain.

## Methods

### Participants

Twenty-four undergraduate students from the University of Gießen participated in our study (17 females and 7 males). All were right-handed by self-report. The students were on average 25 years old (SD = 6 years). All participants were naïve regarding the aims of the study and gave written informed consent prior to the experiment. They were paid 8€ per hour of participation or received course credit. The experimental procedure was approved by the local ethics committee LEK FB06 at Giessen University (proposal number 2009-0008) and in agreement with the Declaration of Helsinki. Due to a technical error in data collection, we had to exclude data from ten participants. This failure led to missing data in some but not all conditions because the Optotrak infrared markers were not visible and the data were hence unusable for the within-subject comparisons. Although there are many possible reasons for missing data, we cannot completely exclude the possibility of a systematic bias, where only a certain type of movement hid the markers from the cameras. However, such systematic dropout would most likely have worked against the hypothesis of finding differences between the conditions. We additionally found a strong correlation between the remaining data of the excluded and the included participants. Here, we will thus present data of the remaining 14 participants.

### Stimuli

**Fig. 1:**
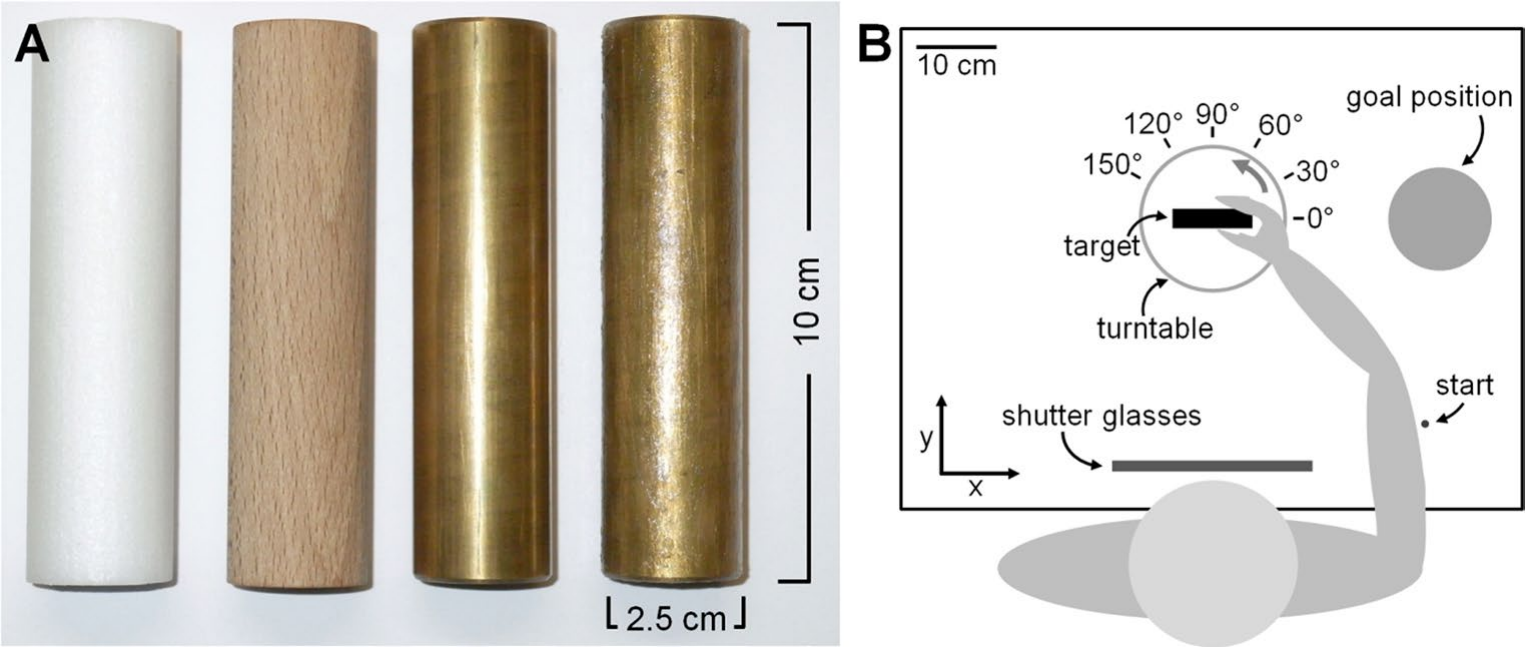
**a** The four equally sized cylinders used as target objects in the experiment: one made of Styrofoam, one made of wood, one made of brass and an additional brass cylinder covered with Vaseline (from *left* to *right*). **b** Sketch of the experimental setup. Participants were seated in front of the table and performed the grasping movements with their right hand. After an auditory signal and the shutter window turning transparent, they moved from the indicated start position to the target object, which could be presented at one of the six different orientations by means of the turntable. They grasped the object with a precision grip, lifted it and carried it to the indicated goal position, where they released the object, and returned to the start position

Four equally sized cylinders served as stimuli in our experiment (see Fig. 1a). The height of the cylinders was 10 cm, and their diameter was 2.5 cm. One cylinder was made of white fine-grained Styrofoam (2 g), one of beech wood (36 g) and one of brass (414 g), and one additional brass cylinder was covered with Vaseline to make it very slippery. Thus, our stimuli had the same size and shape but varied in their material, i.e., their weight and surface properties. The Styrofoam and wooden cylinder were rough, the brass cylinder was rather smooth, and the Vaseline-coated cylinder was very slippery. Hence, weight and surface properties covaried in our stimuli. While this prevents us from completely disentangling their effects, our goal was to investigate ‘realistic’ stimuli in which the object properties naturally covary, rather than ‘illusory’ stimuli in which the properties were artificially placed in conflict.

### Setup

Figure 1b shows the experimental setup. Participants sat in front of a table with their head on a chin rest. A pane of liquid crystal shutter glass (Milgram 1987) was mounted in front of the chin rest so that vision of the stimuli and the arrangement of the setup could be obscured between trials. The cylinders lay on their long side on a turntable that was inset in the table at a distance of 36 cm (from the edge of the table to the center of the turntable and thus also the center of the target object). The turntable enabled us to present the cylinders at different orientations. We used the following angles with respect to the participant: 0°, i.e., horizontally, 30°, 60°, 90°, 120°, 150° rotated counter clockwise. A small plastic knob on the right side of the subject, 36 cm away from the center of the turntable, indicated the start position of the movement. A circular goal location (diameter: 13 cm) was positioned 28.5 cm to the right of the target position (center to center). The surface of this goal location was 3.7 cm above the table surface, ensuring that the participants had to actually lift the object, and a small edge around its circumference so that the cylinders would not roll away once placed on the goal surface. An Optotrak 3020 camera system was positioned diagonally on the left of the setup. To record the grasping movements, a small (approximately 1 cm^2^) rigid body, consisting of three infrared markers, was placed on the nail of index finger and thumb of the participants’ right hand. We calibrated the position of the fingertips in relation to these rigid bodies prior to the experiment. In order to do this, we asked participants to grasp a small plastic object (1.5 × 1.5 × 5.0 cm) with a precision grip at two points whose exact positions had been measured before the experiment. Movement of the fingers was captured at 100 Hz.

### Procedure

Prior to each trial, participants were required to place their index finger and thumb at the start position. As soon as their fingers reached this location, the shutter glass became translucent so the participant could not see the experimenter placing one of the objects at the target position. The start of a trial was signaled by a computer-generated sound (beep) and the shutter glass turning transparent. Participants were instructed to grasp the object with a precision grip using the index finger and thumb of their right hand, lift it, transport it to the goal position and place it there without letting it fall. No further instruction was given on how or where to grasp the target objects or how to place it onto the goal surface. Participants had 3 s to complete a trial before the shutter glass turned white again and data collection was stopped, which provided sufficient time for participants to perform the movement at natural speed. Six seconds were given for the slippery brass cylinder. Trials were repeated if the object fell over; the markers at the fingers did not stay at their position (in this case, the markers were fixed and recalibrated before continuing); or were for some reason not visible to the cameras during the trial. In that case, the given trial was repeated at a random position within the remaining trials. Before the start of the experiment, participants completed five to ten practice trials with a different cylindrical object (a marker pen) to become accustomed to the task. One hundred and twenty trials were then completed by each participant, i.e., 4 materials × 6 orientations × 5 repetitions. Trial order was random with one exception; for practical reasons, the trials with the slippery brass cylinder were blocked (either at the beginning or the end of the experiment, counterbalanced between participants) because the setup as well as the fingers of the participant was covered with Vaseline during and after these trials. To exclude the possibility that the results were influenced by this, we asked three additional naïve participants to grasp all objects in a blocked fashion (with randomized orientation). We found that their data were highly correlated with the data of the main experiment (correlation between 8 of 10 dependent variables were significant, all *p*s < .01, average correlation coefficient: *M* = .72), except for the RTs (*r* = .12, *p* = .59), which seems plausible when comparing random to blocked presentation, and the SD of the height of grasp points (*r* = .08, *p* = .72) which might be explained by a ceiling effect since the variation of grasp points was very low overall because they were physically limited by the height of the objects. Furthermore, based on previous literature (Weir et al. 1991a; Fikes et al. 1994), we concluded that, if anything, blocking that condition would have led to smaller effects of that material compared to the others (the opposite of what we found in our main experiment).

### Data analysis

Movement data from single trials were first analyzed individually to segment the movement based on the following key events. The start of the movement was defined as the point in time at which the hand (i.e., the average of index finger and thumb) exceeded a velocity of 0.025 m/s. Reaction time was defined as the duration between the start of the trial as indicated by the beep (and the opening of the shutter window) and the start of the movement. The time between movement onset and first contact with the object was considered the approach time. The maximum grip aperture was defined as the maximum opening of the two fingers in this time period.

To determine the moment of contact with the object, we used *multiple sources of information* (MSI) as described by Schot et al. (2010). Accordingly, six objective functions were applied to all time frames of every trial and multiplied to a combined function. The maximum of that function was defined as the moment of contact. The six criteria we used were the following: (1) Velocity should be low (*F*_*v*_ = 1 − (*v*/*v*_max_), so that low velocities result in larger values in the range between 0 and 1), (2) the moment of first contact happens early in the movement, (3) the hand position (i.e., the average of the position of the index finger and thumb) is not further away from the object center than 70 mm in the *x*–*y* plane (table), (4) the hand position is not further away from the object center than 25 mm in height, (5) the aperture between index finger and thumb is decreasing, and (6) this decrease in the aperture is decelerating. Trials were discarded from further analysis if we could not determine the moment of first contact using these criteria (1.25 % of all trials). Contact points of both fingers with the object were determined for that moment in time in all three dimensions. The variables of interest were the lengthwise deviation of the grasp center (i.e., the mean position between index finger and thumb) from the object’s center, defined as the Euclidian distance between these two points in the *x*–*y* plane, as well as the crosswise deviation, i.e., the deviation of the grasp center from the object’s center in terms of height (*z*-dimension). Besides the deviations from the center of mass, we were also interested in the variability of grasp points, which we defined as the intra-individual standard deviation of the position of the grasp center in the *x*–*y* plane as well as the *z*-dimension.

The moment of lift was defined as the point in time at which the upward hand velocity exceeded 0.01 m/s after the moment of contact. The time in between these two events we refer to as the handling duration. Object release at the goal location was determined as the first frame in which the hand position in height was not further than 25 mm away from the goal location and the hand position in the *x*–*y* plane was not further than 100 mm away from the center of the goal location. The time between object liftoff and release was defined as the transport duration. The remaining part of the movement back to the start position was not of interest in the current study, so we did not use these data.

We averaged data of single trials for each participant and condition. The influence of the material and object orientation on reaction time, movement duration, handling duration, transport duration, maximum grip aperture (MGA), the timing of MGA, the lengthwise and crosswise deviation of the grasp center from the object’s center as well as its standard deviation were analyzed with 4 (material) × 6 (orientation) repeated-measure ANOVAs. All ANOVAs were corrected for possible violations of the sphericity assumption following the Greenhouse-Geisser correction provided by IBM SPSS.

## Results

### Timing of the movements

**Fig. 2:**
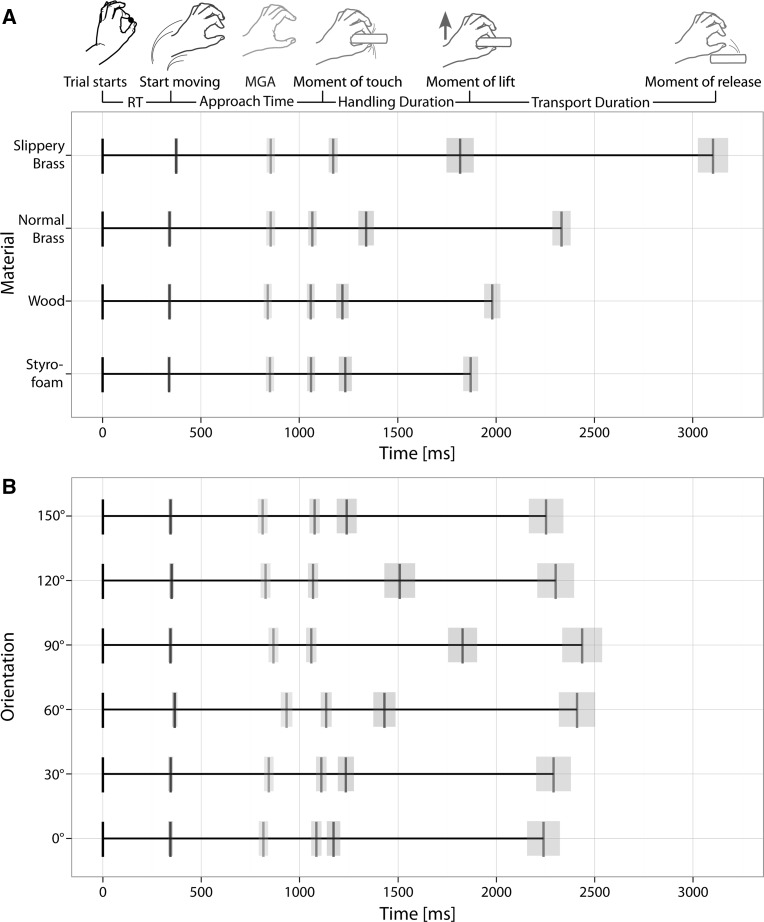
**a** Effects of the object’s material on the timing of the reach-to-grasp movements. **b** Effects of object orientation on the timing of the movement. The *black vertical line* at zero indicates the start of the trial, *blue* the start of the movement, the time in between is the reaction time. *Green bars* mark the moment at which the MGA appeared during the approach movement toward the object, *orange* marks the moment when the fingers first touched the object. Handling duration starts at that moment and lasts until the object is lifted (*red*). This is followed by a transport phase until the object is released at the goal position (*purple*). The *transparent areas* show ±1 SEM between participants at the end of each period for that movement segment, respectively, i.e., the transparent blue bands show ±1 SEM of the reaction time, *green* of the moment of MGA, *orange* of the approach time, *red* of the handling duration, and *purple* bands show the SEM of the transport duration (color figure online)

Figure 2 summarizes the main results of the study in terms of the timing of the reaching, grasping and handling movements for different materials (A) and orientations (B). Mean durations of the individual movement segments are shown one after the other to get an impression of the effects of material and orientation on individual time points as well as the movement as a whole. Differential effects are reported in the following sections.

#### Reaction time

The object’s material had a small but significant effect on reaction time, i.e., the time to initiate the movement after the start signal [*F*(1.41, 18.33) = 6.90, *p* < .05]. Post hoc paired comparisons showed that this main effect was driven solely by the longer reaction times (375 ± 84 ms, mean ± SD) in trials with the slippery brass cylinder (all *p*s < .001, Bonferroni corrected). We found neither an effect of object orientation on reaction times [*F*(2.15, 27.96) = 1.97, *p* = .156, see Fig. 2b] nor an interaction between the two factors [*F*(5.49, 71.31) = 0.88, *p* = .587].

#### Approach time

The time participants took to approach the objects was similarly affected by the material from which the object was made [*F*(1.35, 17.50) = 12.05, *p* = .001]. Participants took longer to approach the slippery brass object (800 ± 155 ms) compared to all other objects (all *p*s < .001, Bonferroni corrected). We also observed a main effect of orientation on the time used to approach the target object [*F*(3.31, 43.01) = 13.21, *p* < .001]. Approaching objects with an orientation of 60° (773 ± 148 ms) took longer than it did for objects with an orientation of 0° (742 ± 143 ms), 90° (717 ± 142 ms), 120° (719 ± 142 ms) and 150° (733 ± 143 ms; all *p*s < .05, Bonferroni corrected). An object orientation of 30° led to significantly longer approaching times (766 ± 143 ms) compared to 90°, 120° and 150° (all *p*s < .05, Bonferroni corrected). There was no significant interaction between two factors [*F*(5.12, 66.72) = 0.74, *p* = .603].

#### Timing of MGA

The MGA occurs during the approach toward the object. Exactly when this maximum opening of the fingers occurred was influenced by the material [*F*(2, 26.04) = 19.57, *p* < .001] as well the orientation of the object [*F*(2.46, 31.91) = 14.44, *p* < .001]. When grasping the slippery brass cylinder, the MGA occurred on average after 72.66 ± 8.42 % of the approach time, which was significantly earlier than with all other materials, i.e., Styrofoam (79.75 ± 7.46 %), wood (78.77 ± 7.17 %) and ‘normal’ brass (79.39 ± 8.42 %, all *p*s < .001, Bonferroni corrected). Note, however, that this effect was not present when considering the absolute time from the start of the movement until the MGA [*F*(1.44, 18.77) = 0.38, *p* = .771], because the approach also took longer in the slippery brass condition. In other words, the MGA occurred on average 576.35 ± 132.37 ms after the start of the movement, irrespective of the material. The MGA occurred significantly later when the target was presented at 60° (81.61 ± 8.47 %) or 90° (81.71 ± 8.57 %) compared to all other orientations, i.e., 0° (74.86 ± 7.72 %), 30° (75.60 ± 7.34 %), 120° (77.03 ± 7.51 %) and 150° (75.02 ± 6.96 %, all *p*s < .001, Bonferroni corrected). There was no interaction between the two factors [*F*(6.38, 82.96) = 0.88, *p* = .521].

#### Handling duration

Larger effects of both material and orientation were observed after participants had made contact with the objects. We found a significant main effect of material [*F*(1.32, 17.18) = 50.45, *p* < .001] and orientation [*F*(2.01, 26.14) = 38.45, *p* < .001] as well as an interaction between the two factors [*F*(3.44, 44.76) = 8.11, *p* < .001]. Time between first contact with the object and liftoff was longer for the slippery brass cylinder (644 ± 600 ms) compared to the normal brass cylinder (272 ± 307 ms), the wooden cylinder (163 ± 219 ms) and the Styrofoam one (174 ± 229 ms; all *p*s < .001, Bonferroni corrected). Additionally, the ‘normal’ brass object was also handled longer before it was lifted than were the wooden and the Styrofoam objects (both *p*s < .001, Bonferroni corrected). The closer to orthogonal the object was positioned with respect to the participant, the longer it took to lift it after first contact. A 90° orientation resulted in a significantly longer handling duration (769 ± 519 ms) than all other object orientations, i.e., orientations of 0° (88 ± 91 ms), 30° (125 ± 164 ms), 60° (295 ± 327 ms), 120° (439 ± 501 ms) or 150° (163 ± 264 ms, all *p*s < .001, Bonferroni corrected). Furthermore, significantly longer handling durations were observed for the 60° and 120° orientation compared to 0°, 30° and 150° (all *p*s < .001, Bonferroni corrected). Object orientation and material also showed interactive effects on handling duration in the sense that the effect of material was more pronounced at orientations that also elicited a larger effect. For example, handling duration was in general longest for the slippery brass cylinder (644 ± 600 ms) and an object orientation of 90° also resulted in the longest handling duration (769 ± 519 ms); a combination of both factors led to an even longer handling duration (1282 ± 695 ms). Thus, the two effects enhanced one another. Figure S1 in the supplementary material shows the handling duration for each of the 24 combinations of our factor levels. It illustrates clearly this superadditivity of our main effects.

#### Transport duration

Similar to the effect on handling duration, we observed a significant main effect of material on transport duration [*F*(1.46, 19.02) = 34.16, *p* < .001]. Transport of the object to the goal position took longer for the slippery brass cylinder (1288 ± 599 ms) than the normal brass cylinder (1000 ± 365 ms), which in turn took longer than the wooden object (765 ± 267 ms) and longer than the Styrofoam object (644 ± 222 ms); differences between all objects were significant (all *p*s < .001, Bonferroni corrected). We also observed a significant main effect of object orientation on transport duration [*F*(2.23, 27.63) = 20.34, *p* < .001]. However, the pattern of results was opposite to what we observed for the handling duration, i.e., here, the closer to orthogonal the object was positioned with respect to the observer, the shorter the transport duration. For an orientation of 90°, transport duration was shorter (619 ± 511 ms) compared to all other orientations, i.e., 0° (1068 ± 426 ms), 30° (1062 ± 429 ms), 60° (978 ± 450 ms), 120° (803 ± 388 ms) and 150° (1014 ± 392 ms, all *p*s < .02, Bonferroni corrected). Similarly, transport from the 120° orientation was significantly faster compared to 0°, 30° and 150° (all *p*s < .01, Bonferroni corrected). There was also a significant interaction effect between both factors [*F*(3.49, 45.33) = 3.45, *p* < .05]. Similar to the interaction effects on handling duration, the effects of the two factors seem to have enhanced one other in a superadditive manner. Figure S2 in the supplementary material shows the transport duration in all 24 conditions.

### Spatial modulation of the movements

Besides the reported temporal effects of material and orientation, we were also interested in the corresponding effects in the spatial domain. This includes the type of grip (i.e., the global configuration of index finger and thumb on the object), as well as the choice of local grasp points and the MGA during the approach toward the object.

#### Grip type

**Fig. 3:**
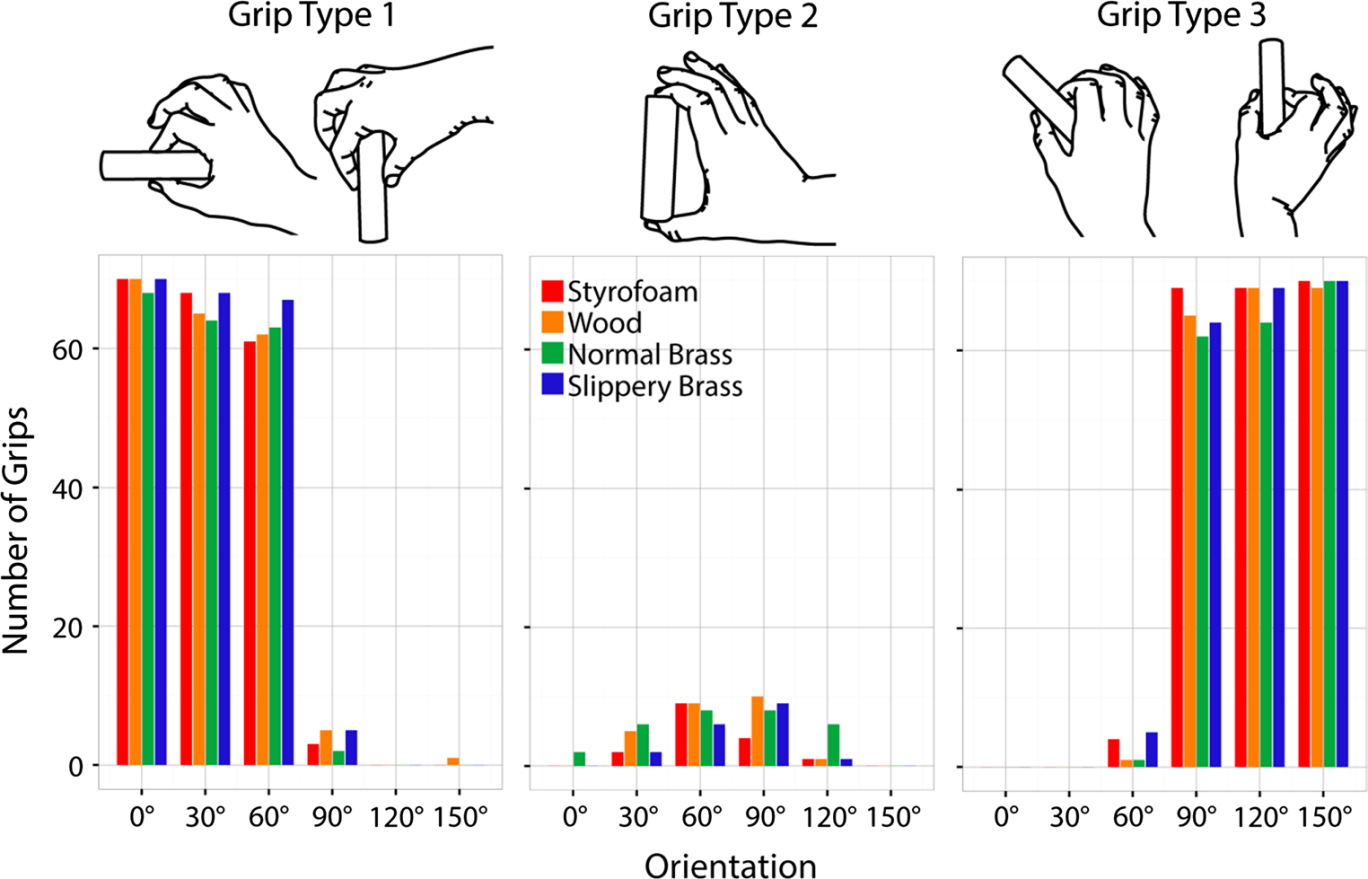
Choice of grip types. *Each plot* shows the absolute frequency of grip types 1, 2 and 3 from *left* to *right*, as a function of object orientation (*x*-*axis*) and material (*color*) (color figure online)

We defined three different grip types, as shown in Fig. 3. In grip type 1, the thumb is on the object side that is closer to the participant when the object is oriented horizontally and rotates in the same manner as the object; at 90° this results in an overhand grip. A grip at the ends of the cylinder was defined as grip type 2 irrespective of the orientation of the object. Grip type 3 refers to grips where at 0° the index finger was on the side closer to the participant and then rotates with the object, resulting in an underhand grip at a 90° object orientation. Figure 3 shows the total number of grip types observed at each orientation for each object. The choice of grip type seemed to be mainly influenced by the object orientation: Grip type 1 occurred almost exclusively at orientations of 0°, 30° and 60°, whereas finger and thumb switched the sides of the objects at 90°, 120° and 150°, resulting in grip type 3. Note that grip types 1 and 3 are similar for more horizontal grip orientations, because the target object was rotated around its own center, i.e., for orientations less than 90°, the thumb is at the closer side in grip type 1, whereas for orientations greater than 90°, it is closer in grip type 3. Thus, in the majority of trials, the thumb was at the side closer to the participant. Fewer trials were observed with a grip at the end of the object (grip type 2); they mainly occurred at orientations where we observed a transition between grip type 1 and 3 (60° and 90°). The object’s material did not seem to have a substantial effect on the type of grip participants selected.

#### Selection of local grasp points

**Fig. 4:**
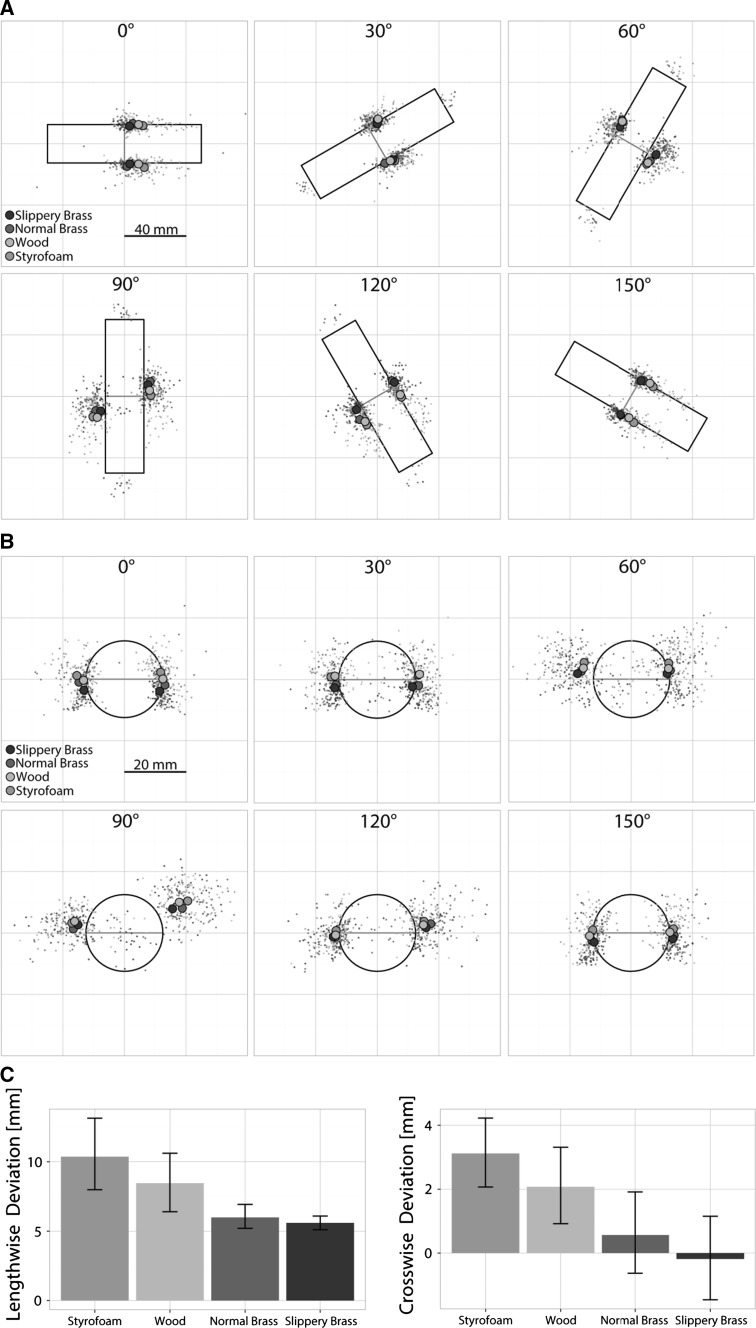
Choice of grasp points on the different objects (*colors*) at different orientations (*subplots*). *Large dots* show the average grasp points; *small dots* show raw data, *gray lines* indicate the position of the COM in one dimension. **a** Plots show a top view of the target object at different angles, i.e., the grasp points in the *x*–*y* plane. **b** Plots show a cross section of the object with corresponding grasp points in the *z*-dimension (height of the object) at different orientations. **c** *Plots* show the mean deviation of the grasp center (average between thumb and index finger) from the COM in the *x*–*y* plane (*left*) and in the *z*-dimension (*right*) for each material averaged across participants and orientations. *Error bars* show ±1 SEM between participants (color figure online)

As well as this more global configuration of the digits during the grasp, we were also interested in how material and object orientation influence the selection and variability of the local grasp points, i.e., where exactly the thumb and index finger touched the object during the grasps. Figure 4 shows the average grasp points (and the raw data) for each object at all orientations.^1^ Figure 4c shows the average deviation from the center of mass (COM) averaged across orientations for the *x*–*y* plane (left side) and the *z*-dimension (right side), respectively. We found a significant main effect of material on the lengthwise deviation from the grasp center to the COM [*F*(1.39, 18.07) = 12.22, *p* = .001], see Fig. 4a, c. Participants grasped significantly further away from the COM when lifting the Styrofoam object (10.4 ± 7 mm, where positive numbers indicate a shift of the grasp center from the COM in the direction of the grasping hand) compared to when lifting ones made of wood (8.5 ± 5.7 mm), brass (6 ± 3 mm) or slippery brass (5.6 ± 2.1 mm, differences between all materials were significant: all *p*s < .01, Bonferroni corrected). Additionally, we observed a significant interaction between material and orientation [*F*(3.15, 40.91) = 5.92, *p* < .001]. The effect of material on the lengthwise deviation of the grasp center from the COM was larger for some object orientations (0°, 120° and 150°) than others. Similar to the other interaction effects, it seems as if the effects of material and orientation might have enhanced each other although object orientation alone did not have an effect [*F*(1.70, 22.09) = 2.12, *p* = .150]. The effect of the object’s material was smaller at orientations that showed a tendency for a smaller effect on their own. This pattern can be observed in figure S3 in the supplementary material.

Participants not only grasped further away from the COM along the object when lifting the Styrofoam or wooden object, and their grasp points were also more variable in that dimension, as indicated by a significant main effect of material on the intra-individual standard deviation of grasp points [*F*(2.34, 30.78) = 5.18, *p* < .01]. Both the normal brass (3.01 ± 2.46 mm) as well as the slippery brass cylinder (2.95 ± 1.37 mm) were grasped with smaller variability than the wooden cylinder (4.17 ± 2.93 mm) and the Styrofoam one (4.19 ± 2.80 mm, all *p*s < .05, Bonferroni corrected). Variability of grasp points was not influenced by orientation [*F*(2.26, 29.36) = 2.79, *p* = .072]. Material and orientation did not interact significantly [*F*(3.67, 47.74) = 2.19, *p* = .089].

The height of grasp points was also influenced by the object’s material [*F*(1.48, 19.28) = 20.26, *p* < .001], see Fig. 4b, c. Grasp points were significantly higher for the Styrofoam object (3.12 ± 3.63 mm, where 0 is the object center and positive numbers indicate an upward shift) compared to the wooden object (2.07 ± 4.00 mm), the normal brass cylinder (0.57 ± 4.37 mm) as well as the slippery brass cylinder (-0.19 ± 4.55 mm). Differences between all objects except normal and slippery brass were significant (all other *p*s < .01, Bonferroni corrected). Also object orientation influenced the height of grasp points [*F*(2.70, 35.05) = 32.23, *p* < .001]. They were higher, the more orthogonally the object was positioned with respect to the participant, i.e., grasp points at 90° (5.98 ± 3.02 mm) were higher than at 60° (3.38 ± 4.32 mm), 120° (1.08 ± 2.66 mm), 30° (-0.38 ± 3.71 mm), 150° (-0.81 ± 3.54 mm) and 0° (-0.89 ± 3.75 mm). Differences between all orientations (except the comparison between 150° with 30° and 0°, respectively) were significant (all *p*s < .05, Bonferroni corrected). There was no interaction effect between both factors material and orientation [*F*(6.53, 84.9) = 2.05, *p* = .62]. There was no significant difference in the intra-individual variability of the height of the grasp center due to material (*F*(2.34,30.91) = 1.93, *p* = .156), orientation [*F*(3.86, 50.24) = 1.01, *p* = .409] or both [*F*(6.53, 84.85) = 1.06, *p* = .393]. It should also be noted that there were also fewer possible grasp points in that dimension, so this might be a ceiling effect.

#### MGA

We excluded trials in which participants grasped the target at its long axis (grip type 2, see above) and as a consequence one participant, because of missing data. We did this in order to avoid introducing ceiling effects. For the remaining trials, we observed no main effect of the different material properties on the MGA [*F*(1.47, 17.64) = 2.19, *p* = .150]. However, orientation did influence the size of the MGA [*F*(1.39, 16.63) = 10.62, *p* < .01]; it was larger for objects with a more orthogonal orientation, i.e., at 90° (50.08 ± 10.71 mm), it was larger than at 0° (40.28 ± 7.49 mm), 30° (40.93 ± 6.34 mm), 60° (43.50 ± 6.54 mm), 120° (44.57 ± 10.20 mm) or 150° (41.48 ± 8.32 mm). Furthermore, the MGA was larger when approaching objects at 60° or 120° compared to 0°, 30° and 150° (all *p* < .05). There was no interaction between the two factors [*F*(6.00, 72.05) = 1.94, *p* = .086].

## Discussion

We found that the spatiotemporal parameters of reaching, grasping and handling movements were systematically influenced by both the material and orientation of the object. Higher weight and lower surface friction increased the duration of individual movement segments, especially after the hand had made contact with the object. Orientations closer to orthogonal (with respect to the participant) led to longer handling durations and shorter transport times. These effects of material and orientation seemed to have enhanced each other interactively. Variation of the spatial layout of the object, i.e., its orientation, had a large effect on the spatial configuration of the grip in terms of the type of grip that was used and the local grasp points chosen on the object as well as on the MGA. Material, on the other hand, only affected the spatial modulation on the smaller scale, i.e., the choice of contact points on the object.

It seems that the materials we used in this experiment impose different requirements on the actor, making the task easier or more difficult to execute. These different demands affect different aspects of the reach-to-grasp movement and thus different measures in our experiment. An object with high surface friction and low weight (such as our Styrofoam object) can be grasped further away from the center of mass and still result in a successful grip (Paulun et al. 2014). Such objects might be grasped with less precision because the costs of grasping off-center are low (in terms of additional forces to counteract accruing torques) as are the risks of the object slipping or rolling out of the hand. Higher precision, on the other hand, would increase the costs in terms of time (although the time constraints were rather low in this task) and effort, i.e., a more thorough planning and execution. This is indeed what we found here. Grasp points were further away from the center of mass and more variable in the *x*–*y* plane for objects that were lower in weight and higher in surface friction. Additionally, we found that grasp points were higher for these objects. Conversely, for heavier objects with less friction, the grasp axis went either through or below the COM. This might reflect a safety strategy which ensures that if the object were to slip in the hand, it would not drop directly onto the table, but instead the fingers would slide below the object and thus hold it in that position. The global configuration of the index finger and thumb during the grasp movement, i.e., the type of grip, was not influenced by the material or if it were, the influence was obscured by the dominant effect of the orientation. Unsurprisingly, this parameter seemed to be largely determined by orientation (see below).

The different demands on grasp precision are also reflected in the timing of the movements, starting from the very first moment the participant sees the object until it is released at the goal location. Thus, we found that even the reaction time for initiating the movements varied for different materials. This effect was driven largely by the slippery brass cylinder: RT was significantly longer when grasping this object, although it should be noted that this object was presented in a blocked fashion. Presumably, this effect reflects a longer planning phase for the upcoming movement. Previous research investigating the effects of different materials on grasping has for the most part not measured RT (or has not differentiated between RT, approach time and handling duration). Only Fleming et al. (2002) reported longer RTs for more slippery objects when there was no time constraint, similar to our task. In our experiment, the approach toward the object also took longer on trials in which the slippery brass cylinder had to be grasped, compared to when other objects were grasped. This might reflect the more thorough planning of the movement as well as a more careful approach to the selected grasp points. A slower approach is a second mechanism that will decrease the variability of grasp points (Fitts 1954). In other words, because grasping the heavy, slippery object requires high precision, participants adopt a slower approach to achieve a different speed–accuracy trade-off. Furthermore, our results are in line with previous research reporting a longer approach toward heavier (Weir et al. 1991b) or more slippery objects (Fikes et al. 1994; Weir et al. 1991b; Flatters et al. 2012) or both (Fleming et al. 2002; Paulun et al. 2014), although most of these studies do not differentiate between RT, approach and handling duration as we do here.

We observed larger effects of the material on movement timing *after* the hand had made contact to the object, i.e., for the handling and transport duration. Handling duration was longer when the object was rather heavy (brass vs. other materials) and slippery (brass covered with Vaseline vs. other objects). This prolonged time until liftoff is probably required in order to estimate and generate the necessary grip and load forces that will assure a stable grip. Johansson and Westling (1984) have shown that the time until sufficient forces are reached increases with an increase in weight of the object to be lifted. Presumably, even more time is required to lift a slippery object because grip forces have to be adjusted more precisely as too little force will not be sufficient to lift the object and too much force will lead to the object slipping in the hand. The longer handling duration we observed for larger weights and lower friction is in line with previous research looking at both features individually (Weir et al. 1991a, b) and might be regarded as a sign for ‘stop’ as opposed to ‘on-the-fly’ grasps as defined by Flatters et al. (2012) and therefore in accordance with the results of their study. For the transport duration, we found significant differences between all objects: The durations were longer, the heavier the object was and the less friction its surface had. A shorter transport duration can be achieved through a sharp acceleration and deceleration during the movement. Larger forces are required to accelerate/decelerate heavier objects, and it is more difficult to maintain a stable grasp during these phases when the object is heavy and/or has less surface friction. This might have led to the effect of material on transport duration we found in this experiment. Since the duration of the transport when grasping objects made of different materials has not been reported before, we cannot compare it to previous literature. For future research, it would be desirable, although technically challenging, to measure not only the kinematics but also the forces that caused the observed motion, especially with regard to the handling and transport phase. This would enable us to better understand the control mechanisms behind the movement. One might, for instance, further understand the trade-off between speed, accuracy and required force, e.g., quantifying the costs of grasping faster, but further off-center (i.e., less accurate) which thus requires more force to lift and hold the object. It might also be possible to disentangle the individual contribution of force generation and other factors to the timing of the movement.

Similar to different materials, different object orientations can also make a grasping movement easier or more difficult. This of course depends highly on the shape of the object to be grasped. Here, we investigated elongated cylindrical objects that were rotated in the horizontal plane. This orientation largely determined the grip type that was chosen, i.e., the orientation of the hand: When the object was presented at 0°, 30° or 60°, the participants almost exclusively chose grip type 1, whereas 90°–150° almost exclusively led to grip type 3. Grip type 2 on the other hand was chosen only very rarely at orientations of 60°–120°. So overall, participants preferred to grasp the objects around their short axis. However, this preference might also have to do with the size of the cylinders. Depending on the size of the participants’ hand (which we did not measure), it might have been uncomfortable or even impossible to grasp the object along its long axis. In general, participants aimed to place the thumb on the side of the object that was closer to the body, and this might be in accordance with their natural grasp axis (Lederman and Wing 2003; Kleinholdermann et al. 2013). Such posture might be preferred because it is within the dynamic range of the hand, in which its pose can easily be adjusted in response to changing requirements or feedback. If, however, the effector is already at the limits of extension at the beginning of the grasp, fewer corrections are possible. For an orthogonal object orientation (or orientations close to orthogonal), this type of grip (with the thumb on the closer side) is not possible and the resulting grip will be less comfortable. Indeed we found most variability of grip types at orientations of 60°–120°. Differences in wrist rotation (which is closely linked to the grip type as defined here) in response to object orientation have also been reported in previous literature (Cuijpers et al. 2004; Desmurget and Prablanc 1997; Fan et al. 2006; Gentilucci et al. 1996; Mamassian 1997). Also with regard to the local grasp points, we found that most differences occurred when comparing orientation close to horizontal versus close to orthogonal. Grasp points were higher for more orthogonal objects. There was no main effect of orientation on the choice of grasp points in the *x*–*y* plane, but the effect of material was larger when the object was closer to horizontal, i.e., with the long edge facing toward the participant. These orientations might be more comfortable and thus allow more influence of other (here internal) factors.

The MGA was larger when the object was oriented closer to orthogonal. This can be interpreted as another indicator that these orientations are more difficult for the actor, because increasing the MGA will increase the precision with which the object will be grasped (due to the more orthogonal approach, Smeets and Brenner 1999). In these cases, the MGA also occurred later during the movement, although this might simply reflect the fact that it takes more time to open the hand wider. In general, similar to the effects of the material, orientation had bigger effects after the hand had made contact with the object, although we also found an effect on the approach time whereby the approach was slower when the object was presented at 30° or 60°. This might be related to the kind of wrist rotation that is executed during grip type 1. From our data, however, it is not clear why this should be the case when rotating the wrist in one but not the other direction. Previous literature has not found the duration of the reach toward the object affected by its orientation (Mamassian 1997). Interestingly, orientation had opposing effects on handling and transport duration. The closer to orthogonal the object lay, the longer the handling duration, i.e., the longer it took to lift the object after the fingers had first touched it. It appears as if more time was required to set up a stable grasp at these rather difficult orientations. However, it seems that once a stable grip had been established, the subsequent transport could then be conducted faster. This might be because fewer online corrections are required in the latter case.

The aim of this study was also to investigate if and how material and orientation interact in their modulation of reach-to-grasp movements. We found no such interaction with respect to RT or approach time. These time segments, however, were also the least affected by both factors on their own. There were also no interaction effects observed for the timing of MGA. We did, however, find interaction effects after participants had made contact with the objects, i.e., in their handling and transport durations. In both cases, it seems as if the extrinsic and intrinsic factors enhanced one other, i.e., the effect of the material was larger at orientations that also elicited a larger effect. Handling duration, for instance, was longest for the slippery brass cylinder and longest for objects presented at 90°; it was even longer if the slippery brass cylinder was presented at 90°. A similar superadditive effect of material and orientation was observed for the transport duration. The lengthwise deviation of grasp points was affected by the material, and this effect was modulated at different orientations. Again, it seems as if orientations that tended to elicit larger effects (although there was no main effect here) would enhance the effects of the material itself. Overall it seems that extrinsic and intrinsic factors interacted on many different levels and thus should not be regarded separately. In case of an interaction, it appears as if these factors enhance rather than weaken or reverse each other.

## Conclusion

As is evident in everyday life, humans are able to adjust their precision grip to the various requirements demanded by different materials and object orientations. Here, we described how the kinematics are adapted in response to systematic variations of these factors. It appears as if a higher weight, lower friction and an object orientation close to 90° with respect to the actor (i.e., the base pointing toward the actor) make grasping more difficult, leading to longer planning and execution phases and more careful placement of the fingers on the object. Both these intrinsic and extrinsic factors influenced the movement after contact with the object was made but also prior to this, emphasizing the role of vision in guiding manipulative actions. How exactly these visuomotor transformations are achieved by our sensorimotor system is a matter for future research.

## Electronic supplementary material

Below is the link to the electronic supplementary material.

Supplementary material 1 (PDF 2180 kb)

## Footnotes

1 Note that as displayed, some contact points appear within the rectangles representing the cylindrical bars in this figure. There are several potential reasons for this: (1) Fig. 4a shows the grasp points irrespective of their height along the cylinder’s short axis, whereas the outlines in the graph show the object only at its widest point (i.e., its center). Thus, if participants grasped below or above the center (which they did, see Fig. 4b), these points will appear within the object in this representation. Similarly, grasp points appear within the cylinder in Fig. 4b when participants grasped the object at its long axis. (2) Grasp points may appear slightly within or outside the object, if participants did not touch the object with the calibrated tip of their finger, but with a point slightly more proximal or distal. (3) Finally, although we carefully measured and analyzed our data, there might still be some residual noise that may have led to outliers.
