# Supplementary material for: Effects of material properties and object orientation on precision grip kinematics
Source: Exp Brain Res. 2016 Mar 26;234:2253–65. doi: 10.1007/s00221-016-4631-7 (PMC4923101; doi:10.1007/s00221-016-4631-7)
Supplement: Supplementary file 1 — Supplementary material 1 (PDF 2180 kb) [file 221_2016_4631_MOESM1_ESM.pdf]

Effects of material properties and object orientation on precision grip kinematics

Supplementary Material

Vivian C. Paulun[1☆], Karl R. Gegenfurtner[1], Melvyn A. Goodale[2], & Roland W. Fleming[1]

1 *Department of Experimental Psychology, University of Gießen, Germany*
2 *The Brain and Mind Institute, The University of Western Ontario, Canada*
☆ *Corresponding author: Vivian.C.Paulun@psychol.uni-giessen.de*

Content

- Interaction effects: Material × Orientation
  - Figure S1: Handling duration
  - Figure S2: Transportation duration
  - Figure S3: Lengthwise deviation from center of mass

Interaction effects: Material × Orientation

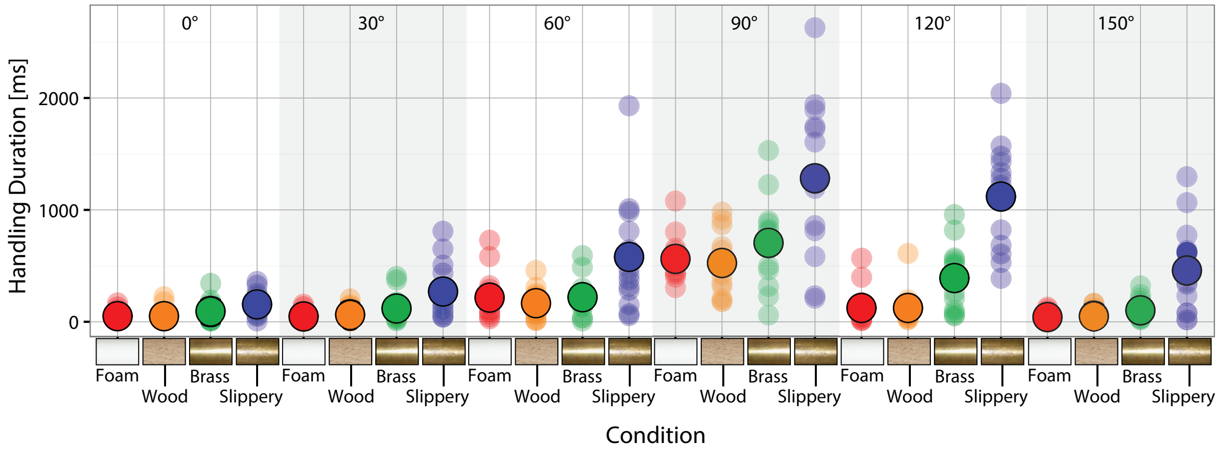

Figure S1. Interaction effects of material (different colors) and orientation (different columns) on handling duration. Large dots with black edging show the mean across participants, semi-transparent dots show the mean of individual participants, averaged across trials. The two factors showed a significant interaction ($F(3.44, 44.76) = 8.11$, $p < .001$) that appears to be in a superadditive manner.

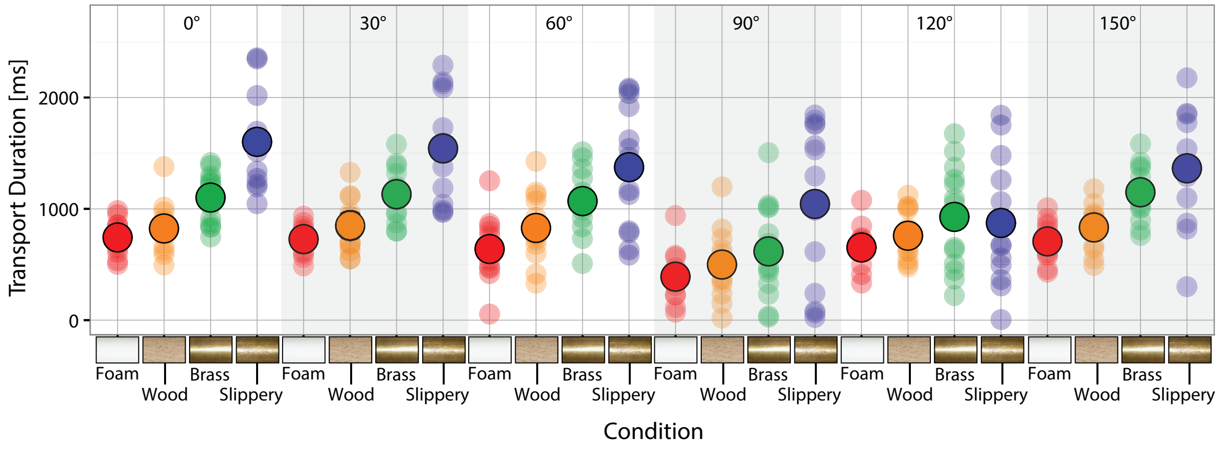

Figure S2. Interaction effects of material (different colors) and orientation (different columns) on handling duration. Large dots with black edging show the mean across participants, semi-transparent dots show the mean of individual participants, averaged across trials. The two factors showed a significant interaction ($F(3.49, 45.33) = 3.45$, $p < .05$). Similar to the effects on handling duration, the two main effects seem to be superadditive.

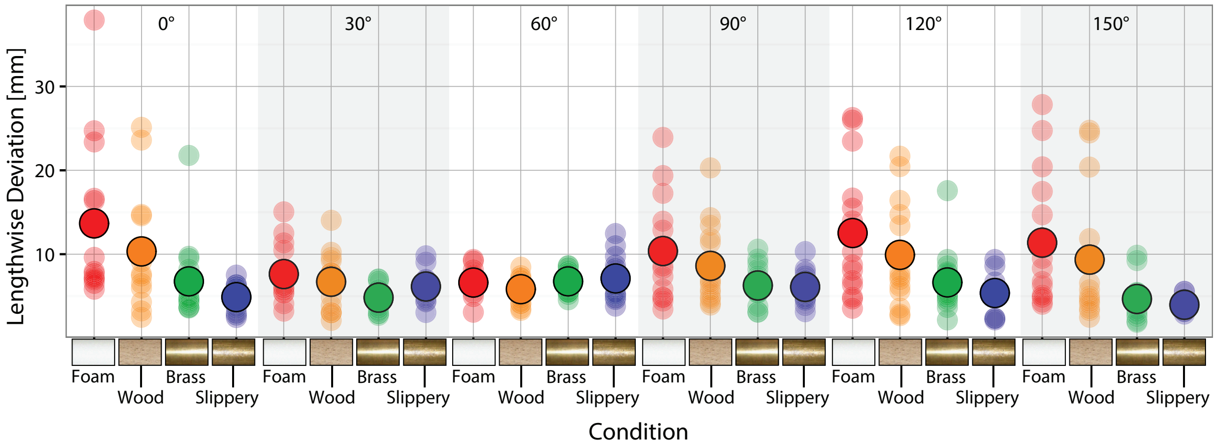

Figure S3. Interaction effects of material (different colors) and orientation (different columns) on the lenghtwise deviation of the grasp center from the object's center of mass (COM). Large dots with black edging show the mean across participants, semi-transparent dots show the mean of individual participants, averaged across trials. Material and orientation showed a significant interaction ($F(3.15,40.91) = 5.92$, $p < .001$): The effect of material was more pronounced at some angles than at others.
